# Comparative Reduction of Egg Yolk Cholesterol Using Anionic Chelating Agents

**DOI:** 10.3390/molecules23123204

**Published:** 2018-12-05

**Authors:** Minerva Bautista Villarreal, Claudia T. Gallardo Rivera, Eristeo García Márquez, José Rodríguez Rodríguez, María Adriana Núñez González, Abelardo Chávez Montes, Juan G. Báez González

**Affiliations:** 1 Departamento de Alimentos, Facultad de Ciencias Biológicas, Universidad Autónoma de Nuevo León, Av. Pedro de Alba s/n, Cd. Universitaria, C.P. 66455 San Nicolás de los Garza, Mexico; minerva.bautistavl@uanl.edu.mx (M.B.V.); claudia.gallardorv@uanl.edu.mx (C.T.G.R.); maria.nunezgn@uanl.edu.mx (M.A.N.G.); 2 Centro de Investigación y Asistencia en Tecnología y Diseño del Estado de Jalisco, A.C. Unidad Noreste, Parque PIIT, vía de la innovación 404, C.P. 66600 Apodaca, Mexico; egarcia@ciatej.mx; 3 Instituto Tecnológico de Estudios Superiores de Monterrey (ITESM), Escuela de Ingeniería y Ciencias, Av. Eugenio Garza Sada 2501 Sur, Col. Tecnológico, C.P. 64849 Monterrey, Mexico; jrr@itesm.mx; 4 Departamento de Química, Facultad de Ciencias Biológicas, Universidad Autónoma de Nuevo León, Av. Pedro de Alba s/n, Cd. Universitaria, C.P. 66455 San Nicolás de los Garza, Mexico; abelardo.chavezmn@uanl.edu.mx

**Keywords:** egg yolk, cholesterol extraction, granules extraction, anionic chelating biopolymers

## Abstract

Egg yolk is used as an emulsifying agent. Nevertheless, its high concentration of cholesterol is linked to chronic degenerative diseases that cause cardiovascular disease. In this study, three methods for reducing the level of cholesterol in egg yolks were studied. The first method consisted of physical separation of the granules contained in the yolk (Na_G_). The second method applied was the use of anionic chelating biopolymers, such as arabic gum solution (AG) and mesquite gum solution (MG), and the third method was extraction with a solvent (S_A_). For this purpose, the cholesterol present in egg yolks, the microstructure, particle size, zeta potential, and its emulsifying capacity were determined. The amount of cholesterol removed was 97.24% using 1% mesquite gum (MG_1%_), and 93.26% using 1% Arabic gum (AG_1%_). The zeta potential was determined, and the isoelectric point (*ζ* = 0) of egg yolk was identified as pH 4.6. While, at this pH, the zeta potential of mesquite gum was −14.8 mV, the zeta potential for the arabic gum was −16 mV. The emulsifying capacity of MG_1%_ was 62.95%, while the emulsifying capacity of AG_1%_ was 63.57%. The complex obtained can be used in the development of functional foods reduced in cholesterol.

## 1. Introduction

Egg yolk is a good source of lutein, zeaxanthin, proteins, lipids, and vitamins in human nutrition and is made up of practically 50% solids. The major constituents of the solid matter are lipids (65–70% on dry basis) and proteins (30% on dry basis). The proteins present are livetins, lipoproteins [1], and some particles including high-density lipoproteins (HDLs), low-density lipoproteins (LDLs), and phosvitin [2,3].

Egg yolk is an efficient ingredient in many food products, and its functional properties include emulsifying, coagulating, foaming, and gelling properties [4]. Moreover, it contains proteins, vitamins, minerals, essential fatty acids, phospholipids, and other compounds. However, it has high cholesterol content; one simple egg contains between 200 mg and 300 mg of cholesterol/100 g; therefore, it almost meets the dietary intake limit set by the American Heart Association of <300 mg/day [3,5]. Consuming products with a high amount of cholesterol can result in cardiovascular disease. Clinical studies demonstrate that dietary cholesterol may increase serum LDL in certain individuals (hyper-responders). This is generally accompanied by an increase in HDLs [6]. Sichittiano et al. [7] undertook a review of nutraceuticals and functional food ingredients that are beneficial to vascular health. Grape seeds can reduce blood lipid levels, since it includes proanthocyanidins (polyphenols), which seem to play the main role in this process. Proanthocyanidins reduce the levels of triacylglycerol in chylomicrons and in very-low-density lipoproteins (VLDLs) [8]. Other functional ingredients are anthocyanins, which act on LDLs and HDLs. The influence on the lipid profile of anthocyanin supplements obtained from berries was evaluated in dyslipidemic patients. A decrease in LDLs was observed in patients after 12 weeks of treatment [9]. Mesquite gum has some phenolic compounds that are trapped in the gum matrix, and these substances are involved in the defense of the plant; in addition, the polymerization of these compounds produces polyphenols, resulting in brown or yellow gum [10].

Several researchers worked on different ways of decreasing the amount of cholesterol present in egg yolks. Warren et al. [11] used solvents such as hexane. Hexane forms a blend with egg yolk solids, but it requires a process of filtering to remove the solvent and a prolonged drying period. The yield reported was 62.2% cholesterol. Borges et al. [12] used a ratio of 1:12 *w*/*w* (yolk/acetone), and the emulsifying properties were maintained.

Paraskevopoulou et al. [13] extracted cholesterol from egg yolk with ethanol/water 20:80 (*v*/*v*), and 1.5% (*w*/*v*) polysorbate 80; after that, the dispersion was then centrifuged, and the yolk precipitate had 7.1 ± 0.3 mg of cholesterol. Laca et al. [14] worked on the extraction of egg yolk granules. In this process, egg yolk was mixed with water (1:15 *v*/*v*), the pH was adjusted to 7, and it was kept overnight at 4 °C; the granules had a concentration of 291 mg of cholesterol/100 g of egg yolk, equivalent to a reduction of 77% cholesterol. Another method involved the reduction of cholesterol using β-cyclodextrin (β-CD) due to its affinity for non-polar molecules such as cholesterol [15]. Jeong et al. [16] reported that cholesterol removal was 92.76% when using 25% crosslinked β-CD at 40 °C. Chiu et al. [17] used the immobilization of β-cyclodextrin in chitosan beads (Ch-BCD) by cross-linking with 1,6 hexamethylene diisocyanate (HMDI) reagent for cholesterol absorption from egg yolk, removing 92% of the cholesterol. That cholesterol was removed using 1% *w*/*v* Ch-BCD for 2 h at a proportion 1:30 yolk to water, and a mixture of β-cyclodextrins with chitosan. Garcia et al. [18] used high-methoxyl pectin at a concentration of 3% *w*/*w*, ionic strength 0.39 M, and pH equal to 9.2, and subsequently obtained a reduction of 88.6% cholesterol. Hsieh et al. [19] developed a complex of egg yolk and acacia gum applying the following concentrations: 1%, 3%, 5%, and 10% (*w*/*w*) with cholesterol extraction rates of 70%, 86%, 79%, and 59%, respectively.

Meanwhile, anionic biopolymers, such as gum ghatti, gum tragacanth, gum karaya, xanthan gum, and especially, Arabic gum (AG), are chelating agents, forming complexes with lipoproteins in the yolk. Lipoprotein molecules are positively charged, and anionic polysaccharides are used as chelating agents, controlling pH and temperature [20].

The methods mentioned above for removing cholesterol are complicated because solvents are used, thus prolonging the extraction time. It was shown that the use of biopolymers, including commercial arabic gum, decreases the concentration of cholesterol present in the egg yolk. However, the shortage of arabic gum, due to drought in the regions where it is produced, stimulated the search for other botanical sources that offer greater security of supply and costs [21]. Therefore, the use of mesquite gum as an alternative to arabic gum is proposed in this study. Mesquite gum (MG) has an ability to stabilize colloidal particles (1–100 µm), which it disperses or emulsifies. This property is manifested due to protein arabinogalactans, which allow adsorption in liquid–liquid interfaces [22,23]. The aim of this work was to reduce the amount of cholesterol present in egg yolk by preparing a complex of biopolymer mesquite gum–yolk and arabic gum–yolk, before comparing the results with cholesterol extraction using a solvent, and with physical separation of the granule using sodium chloride.

## 2. Materials and Methods

### 2.1. Materials

Eggs were purchased from the local market (commercial brand name “El Dorado”; a box containing 30 white eggs, expiration date 9 April 2018, batch 043501). Salt, sugar, and vinegar were purchased at the supermarket. The separation of the yolk from albumen was undertaken manually. The vitelline membrane was then cut with a scalpel blade, and the content of the yolk was collected in a glass vessel. The yolk was then mixed gently with a glass rod. The solution was maintained at 4 °C in refrigeration (pH 7). The arabic gum and mesquite gum were purchased from Natural Products of Mexico (Yautepec, Morelos, México), and the gum was purified according to the method of Vernon et al. [23]. Sodium chloride, acetone, ethanol, and hexane reagent-grade chemicals were purchased from Desarrollo de Especialidades Químicas (Monterrey, Nuevo León, México); the cholesterol standard was obtained from Sigma-Aldrich Chemical (Toluca, México). Deionized water was used in all the experiments.

### 2.2. Cholesterol Reduction in Egg Yolk

The egg yolk was treated with physical separation, polysaccharides (arabic gum and mesquite gum), and a solvent for reducing the cholesterol content. The first process was the physical separation of the granules contained in the yolk (Na_G_) using aqueous salt solution and separation through centrifugation. Another process was the separation of cholesterol using complexation with biopolymers, arabic gum (AG) and mesquite gum (MG). In the third method, acetone extraction (S_A_) was used.

#### 2.2.1. Egg Yolk Granule Extraction (Na_G_)

The cholesterol was removed using the method of Laca et al. [14], with a few modifications; 8.5 g of egg yolk and 11.4 g of 0.15 M NaCl solution (1:1.34) was mixed with a vortex for 1 min at 25 °C. The content was centrifuged (Hermle Labnet Z326, Labnet International, Inc., Wehingen, Germany) at 10,000× *g* for 45 min at 25 °C. Finally, the compounds were separated carefully from the aqueous fraction through decantation. The product obtained was lyophilized, and was stored at −20 °C until analysis with GC.

#### 2.2.2. Anionic Polysaccharide/Egg Yolk Complexes

Complex formation was obtained based on the methodology reported by Hsieh et al. [19]. Stock solutions of arabic gum were prepared at 1% (AG_1%_), 3% (AG_3%_), and 10% (AG_10%_), in addition to 1% (MG_1%_), 3% (MG_3%_), and 10% (MG_10%_) mesquite gum. All solutions were maintained in constant agitation all night long. All concentrations are given in ratios of weight/weight (*w*/*w*).

Firstly, 3 g of egg yolk was mixed with 1 g of gum solution (at the concentrations mentioned above) and 4 g of water. The solution was mixed for 1 min in a vortex (Mixer Labnet International, Edison, NJ, USA); it was then centrifuged (Hermle Labnet Z326, Labnet International, Inc., Wehingen, Germany) at 6000× *g* for 15 min at 25 °C. After that, the supernatant was decanted, and then aggregated with 0.5 g of solution (0.9 M NaCl); this was mixed for 1 min in the vortex. Then, 6 g of ethanol was poured and mixed in the vortex for 1 min at 25 °C, and centrifuged at 6000× *g* for 15 min. After that, the solution was decanted; 6 g of ethanol was added to the lipoprotein/anionic biopolymer complexes, and the solution was mixed, before being carefully decanted. The precipitate complex was quantified [19]. Samples were lyophilized and stored at −20 °C until analysis with GC.

#### 2.2.3. Solvent Extraction 

Extraction of cholesterol with a solvent (S_A_) was performed using the method described by Borges et al. [12] using a ratio (*w*/*w*) of 1:12 (yolk/acetone), and mixing at 100 rpm for 2 min in the stirrer (EURO-ST 60 D S001, IKA, Wilmington, NC, USA). This permitted the separation of the sample after 10 min, and the solvent of the precipitate was carefully decanted. Finally, the precipitate was washed with water. The samples were lyophilized and stored at −20 °C until analysis with GC.

### 2.3. Quantification of Cholesterol Using Gas Chromatography

Method 26.052 of the Association of Official Agricultural Chemists (AOAC) [24] was used. For the acid hydrolysis, 0.2 g of the sample was mixed with 2 mL of methanol, and 7% H_2_SO_4_ (*v*/*v*). Next, the sample was heated for 90 min at 80 °C; after that, the sample was cooled at 25 °C. Then, 3 mL of hexane was added and mixed for 1 min in the vortex (Mixer Labnet International, Inc., Edison, NJ, USA). The solution was kept for 15 min until the formation of two phases was completed; the process was performed twice. The supernatant recovered was mixed and diluted into a 10-mL flask with hexane. Subsequently, the solution was analyzed with GC (7890B, Agilent Technologies, Santa Clara, CA, USA), coupled to a mass spectrometer (5977A, Agilent Technologies, Santa Clara, CA, USA), and equipped with an HP-5MS capillary column (length: 30 m; inner diameter (ID): 0.25 mm; film thickness: 0.25 µm). The injected sample was 1 µL on split mode. The chromatographic conditions were as follows: column temperature 70 °C, kept for 1 min; increased to 200 °C at 10 °C/min, maintained for 2 min; increased to 300 °C at 10 °C/min, maintained for 7 min. The temperature of the injector was 250 °C, and the temperatures of the ion source and quadruple were 230 °C and 150 °C, respectively. The carrier gas helium flow rate was 1 mL/min. The ionization with electron impact was 70 eV and the scan acquisition mode had a range of 30 to 400 UMA. The calibration curve was done with a cholesterol standard of 20 to 120 ppm.

### 2.4. Particle Size Measurement

The particle size distribution of the samples (AG_1%_, AG_3%_, AG_10%_, MG_1%_, MG_3%_, MG_10%_, S_A_, and Na_G_), and the yolk were monitored using a Malvern Mastersizer 3000 (Malvern Instruments, Ltd, Worcestershire, UK) particle size analyzer with a unit of Hydro LV with water as a dispersant. The angular scattering intensity data were analyzed to calculate the size of the particles, creating a scattering pattern using the Mie theory of light scattering. The software calculated the particle size distribution (D_(3,2)_). Optical properties of the sample were defined as a refractive index 1.460 and an absorption of 0.1.

### 2.5. Zeta Potential (ζ)

The zeta potential was determined using dynamic light scattering equipment Zetasizer Nano ZS90 (Malvern Instruments, Worcestershire, England, UK). The measurements were carried out using a universal dip cell (ZEN 1002, Malvern Instrument, Worcestershire, UK) at 25 °C, using the diluted solutions. The zeta potential is related to the velocity of the solutions in an electric field. The equipment software converts the electrophoretic mobility measurements into zeta potential values using the Smoluchowski model [25]. The zeta potential of egg yolk/polysaccharide solutions at different pH levels was measured with the method of Navidghasemizad et al. [26]. About 0.1 g of sample was diluted to a final volume of 20 mL using distilled water, and the pH was adjusted to values of 2.0, 3.0, 4.0, 5.0, 6.0, 7.0, 8.0, 9.0, and 10.0 using 0.1 M HCl or 0.1 M NaOH solution while stirring the samples. Both complexes, yolk/AG and yolk/MG, were monitored for the zeta potential. The zeta potential was calculated from the average of three measurements of the diluted solutions.

### 2.6. Emulsifying Capacity

Samples were prepared individually, and 10 g of sample was mixed (Na_G_, S_A,_ AG_1%_, and MG_1%,_ samples with high yield) with salt (1.59%), sugar (1.06%), and water (11%), separately. Then, the vinegar (3.17%) was added and kept under constant stirring at 300 rpm in the stirrer (IKA Eurostar 60 digital) for 10 min. The test ended when it was not possible to integrate more oil contained in the burette (***V_oil_***), and a layer was observed on the surface of the emulsion (***V_Emulsion_***) [27]. The values were estimated as percentage of emulsified oil (%EC) in total emulsion using Equation (1).

(1) $$\%EC=\frac{V_{oil}}{V_{Emulsion}}\times 100$$

### 2.7. Microstructure Analysis

The topology was analyzed employing scanning electron microscopy (SEM) obtained with the methodology reported by Valverde et al. [28]. Briefly, the samples were fixed overnight in 3% glutaraldehyde in 25 mM phosphate buffer (pH 3.25). After that, the samples were consecutively dehydrated with 20%, 40%, 60%, 80%, and 100% ethanol. Then, the ethanol was consecutively removed with 20%, 40%, 60%, 80%, and 100% acetone, and the samples were analyzed. Finally, they were dried at a pressure of 1 × 10^−2^ Torr (soft vacuum) in a vacuum desiccator. The dried sample was placed on aluminum SEM stubs and coated with gold/palladium. The microscope used was a JSM-6490LV (JEOL, Tokyo, Japan).

### 2.8. Color Analysis

The granules were measured for color in the lightness L*, redness (a*)*,* and yellowness (b*) system. Measurements were carried out using a ColorFlex EZ (Hunter Lab, Reston, VA, USA). A fixed amount of sample was poured into the measurement cell, and analyses were conducted in specular exclusion mode.

The color changes are expressed as **Δ*E*** with the color of the egg yolk as a reference sample [29]; hence, **Δ*E*** is the total color change due to the different contributions calculated using Equation (2).

(2) $$\Delta E=\sqrt{{\left(\Delta L^{*}\right)}^{2}+{\left(\Delta a^{*}\right)}^{2}+{\left(\Delta b^{*}\right)}^{2}}$$

### 2.9. Statistical Analysis

All tmeasurements were performed in triplicate, and ANOVA was performed with a confidence level of 95% (*p* < 0.05) using SPSS 20 software (IBM, SPSS Inc, Chicago, IL, USA). To determine the statistically significant difference between values, a one-way variance analysis and a Tukey test were performed.

## 3. Results and Discussion

### 3.1. Cholesterol Removal in Egg Yolk

The process of extracting egg yolk granules, and chelating with arabic gum and mesquite gum was used as an alternative to preparing yolk with a high concentration of protein and low cholesterol as a functional ingredient in the food industry, especially due to its functional attributes, such as foaming capacity, high level of phosvitin and high-density lipoprotein, and emulsifying and binding properties [28,30]. Notwithstanding the process, other properties, such as the emulsifying of oil, are reduced. In order of importance, the removal of cholesterol from lower to highest was Na_G_ < S_A_ < AG < MG. The cholesterol removed from the egg yolk with Na_G_ (Table 1) was the least effective method (51.43%). It was previously reported that the Na_G_ method with ionic solvent removed cholesterol and increased the protein content. The method is relatively fast and inexpensive. The results obtained in the laboratory were compared with those obtained by Strixner and Kulozik [31]. Based on our data, the removal of cholesterol with solvent was the second best method for cholesterol removal (64.15%). However, Martucci and Borges [32] reported a six-stage extraction system of 92% cholesterol removal in a computer simulation study.

The removal of cholesterol using acetone in the laboratory was lower than the 81%, as reported by Borges et al. [12]. The removal of cholesterol was lower due to the concentration of water in the fresh yolk, the extraction time, and the acetone ratio. It was reported that the use of non-polar organic solvents prevents protein denaturation, especially in emulsifying activity, which is related to the solubility of the protein. Meanwhile, polar organic solvents reduce the emulsifying activity because they break the hydrophobic interactions between lipids and proteins. Even so, the use of solvents is not completely accepted due to the residues that may remain in the product [33].

The use of arabic gum allowed cholesterol removal between 83.85% and 93.26% (Table 1). The concentrations AG_3%_ and AG_10%_ show significant differences (*p* < 0.05). The removal of cholesterol was greater when 1% arabic gum was used. Meanwhile, when 3% and 10% arabic gum was used, the cholesterol removal was almost 83.85% and 89.93%, respectively, despite the fact that pH values were similar to neutral. The structure of arabic gum is a branched heteropolysaccharide with anionic properties. The quantity of arabic gum–yolk depends on the polyanionic properties of the gum, especially of residues of d-glucuronic acid (~2%) and d-glucuronic acid (~21%) [2,34]. The arabic gum has properties of anionic polysaccharides, which may be used as chelating agents, forming insoluble electrostatic complexes (chelating agent/lipoprotein) [18]. Navidghsemizad et al. [35] used a ratio of 50 g of fresh yolk per gram of arabic gum to observe the separation of phases at different pH levels. They concluded that the nature of the polysaccharide and pH had important effects, resulting in the phase separation behavior. 

Other anionic polysaccharides were used as chelating agents [19]; however, their low solubility and high viscosity reduce their practical applications. Mesquite gum is a polysaccharide which contains acidic residues of β-d-glucuronic and 4-*O*-methyl-β-d-glucuronic, bound to mono-sugars or oligosaccharide chains [36,37]. The removal of high cholesterol levels when the mesquite gum solution was used was 1% *w*/*w* (*p* < 0.05). From mesquite gum MG_1%_, MG_3%_, and MG_10%_, extractions of cholesterol of 97.24%, 96.68%, and 96.60%, respectively, were obtained. No significant differences were observed in the different concentrations where mesquite gum was used.

In light of our results, it can be stated that sodium chloride or acetone have a lower capacity to remove cholesterol compared to both anionic polysaccharides. The greater efficiency in cholesterol removal was obtained with mesquite gum at 25 °C. Nonetheless, obtaining complexes from mesquite gum–yolk allowed a high removal of bound cholesterol. We think that the complex obtained can be used as functional supplement, necessary for reducing the unwanted effects of cholesterol. Scicchitano et al. [7] mentioned the importance of reducing lipid levels, especially for coronary artery disease.

### 3.2. Particle Size Measurement

**Figure 1:**
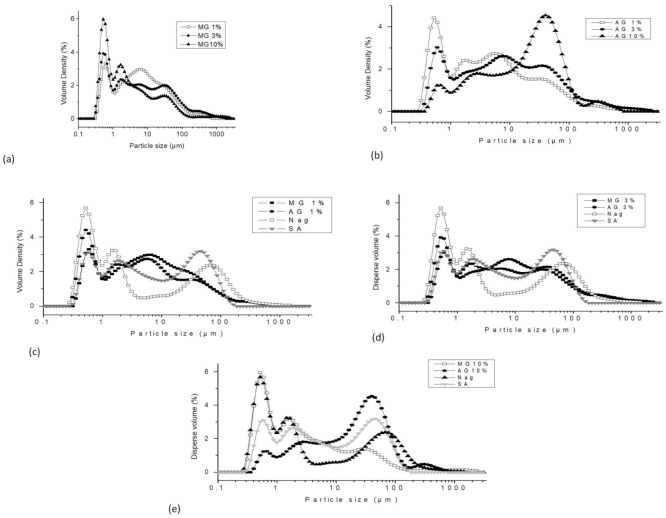
The average particle size: (**a**) −☐− MG_1%_, −●− MG_3%_, and −▲− MG_10%_; (**b**) −☐− AG_1%_, −●− AG_3%_, and −▲− AG_10%_; (**c**) −■− MG_1%_, −●− AG_1%_, −☐− Na_G_, and −▼− S_A_; (**d**) −■− MG_3%_, −●− AG_3%_, −☐− Na_G_, and −▼− S_A_ and (**e**) −☐− MG_10%_, −●− AG_10%_,−▲− Na_G_, −▼− S_A_. MG—mesquite gum; AG—arabic gum; Na_G_—physical separation of granules; S_A_—solvent extraction.

In natural conditions, the yolk is a supramolecular assembly of lipids and proteins, and a highly organized system with approximate size between 0.8 μm and 10 μm [38]. The insoluble structure of yolk has a size range between 0.3 μm and 2 μm [14]. Molecular assembly can be disorganized into individual structures depending on the affinity of the solvent or polysaccharide used to remove cholesterol. The morphology and topology of the particle size determined using the light scattering method can be seen in Figure 1. Figure 1a,b show the granules and the complexes formed with arabic gum and mesquite gum (1%, 3%, and 10%), respectively.

Regarding the distribution of proteins, the structure of the supramolecular system contains many particles of different sizes, three of which are of special interest: the HDLs, which range from 7 to 20 nm; the micelles formed by LDLs in the egg yolk plasma, which range from 17 to 60 nm; and the LDL sources present in the yolk, which range from 80 to 350 nm [31,38]. Anton [39] and Hsieh et al. [19] mentioned that granules of yolk are composed of 70% HDLs, 16% phosvitin, and 12% LDLs. We believe that the granules obtained from solvents and polysaccharides follow the same distribution pattern. The granules and lipoprotein/anionic polysaccharide complexes had different particle size population profiles; these size profiles may be associated with the process of cholesterol removal, the anionic polysaccharides (arabic gum and mesquite gum), and concentration (Figure 1a,b; 1%, 3%, and 10%), and the ratio. The change in distribution of particle size may be due to the viscosity and the charge density of proteins diverse in egg yolk, between MG–yolk and AG–yolk complexes, including the concentration of reactive groups contained in both biopolymers, which form an electrostatic complex [40]. In macroscopic terms, the distribution of granules was separated into three different sizes, based on the chelate concentration. The concentration of chelating polysaccharides at 10% (arabic gum and mesquite gum) had a range of particle size distribution between 0.3 μm and 600 μm. Similarly, the same profile was obtained with low-molecular-weight chelates. The yolk–chelate ratio of 3% for both polysaccharides produced a range of granule size distribution from 0.3 μm to 300 μm (Figure 1d). Finally, when a high molecular weight at 1% concentration was used, the yolk–chelate reduced the size of granules to between 0.3 μm and 250 μm (Figure 1c). More specifically, the granules and lipoprotein complexes obtained had multimodal distributions, as described below. Figure 1e shows three different population distributions when Na_G_, S_A_, and AG and MG at 10% were used. The first range was from 0.3 μm to 1 μm, the second was from 1 μm to 4 μm, and the third range of distribution was from 10 μm to 300 μm, when using the Na_G_ method. The removal of cholesterol using acetone had three different populations of granule size; the range of least distribution was between 0.3 μm and 0.9 μm, and the greatest range showed a variation 0.9 μm to 10 μm. Finally, particle size distribution could be observed in the range of 10 μm to 200 μm. A similar range of distribution was observed when using concentrations of 3% and 1% (both polysaccharides and solvents).

### 3.3. Zeta Potential (ζ)

The interaction between biopolymers may be segregative, due to steric repulsion or associative interactions such as hydrophobic interactions and hydrogen bonding [41]. Electrostatic interactions are the most common force for the complex formation [42]. The pH affected the charge of biopolymers and proteins of the egg yolk, which influenced the zeta potential *(ζ)* as a function of pH; AG_3%_ and MG_3%_ were studied in the pH range of 2–10.

**Figure 2:**
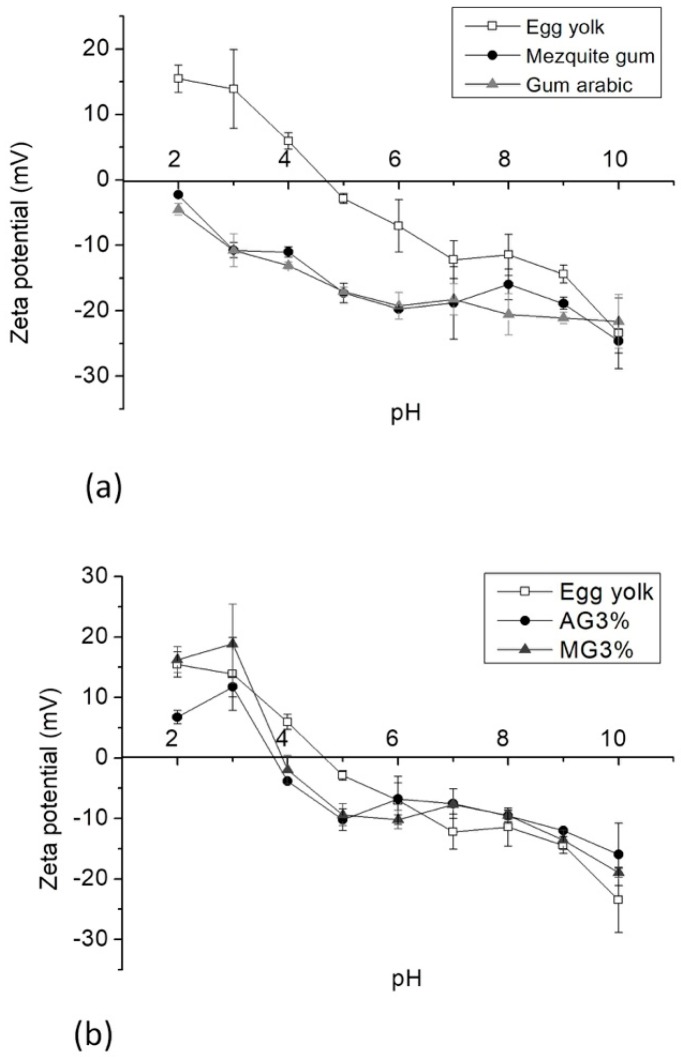
Average zeta potential *(ζ)* of (**a**) −☐− egg yolk, −●− mesquite gum, and −▲− arabic gum, and (**b**) −☐− egg yolk, −●− AG_3%_, and −▲− MG_3%._

The zeta potential of egg yolk was positive at pH values of 2 and 4 (+15.5 mV and +8.5 mV, respectively), while it was negative at pH values of 5 and 10 (Figure 2a); this was similar to the report by Navidghasemizad et al. [26], who obtained positive values in the range of pH 3–5, and, at pH 6, it was negative above the zeta potential. The isoelectric point (*ζ* = 0) of egg yolk was found to be pH 4.6, determined from the zeta potential. While, at this pH, the zeta potential of mesquite gum was −14.8 mV, the zeta potential for the arabic gum was −16 mV. The formation of insoluble complexes appears to occur at pH 3. At this point, the density of the opposite charge between egg yolk and polysaccharides (arabic gum and mesquite gum) has practically the same magnitude. However, the percentage of cholesterol removal was lower than at pH 7. The values are shown in Table 1. The mesquite gum had values of −2.25 mV to −24.61 mV, in the acidic to basic (2 to 10) pH range. We believe that the free and exposed glucuronic acid and protein residues present in mesquite gum reacted in the different media [43] with acidic residues present in the polymer, similar to arabic gum [1]. In Figure 2b, the zeta potential profile of the yolk–3% polysaccharide complex essentially modified the isoelectric point to pH 4 for both biopolymers. For values lower than pH 4, the load profile was positive; however, for values higher than pH 4, the profile was negative.

### 3.4. Emulsifying Capacity

The emulsifying properties only work in specific cases; these properties cannot be generalized. Therefore, we determined the emulsifying properties of the granules obtained, depending on the different treatments. As shown in Table 1, when comparing the emulsifying capacities of the methods discussed, the egg yolk method supports the highest percentage of oil, followed by Na_G_, which is similar to the findings obtained by Laca et al [14]. In AG_3%_, a lower emulsifying capacity than Na_G_ was obtained. It was reported that this is because using arabic gum to remove cholesterol allows the loss of 66% of proteins, including the yolk’s emulsifying proteins, along with lipids [18].

For the egg yolk–mesquite gum complex, the emulsifying capacity of MG_3%_ was 62.95%. It is possible that the ethanol used in the washing of biopolymer–yolk complexes dissolves phospholipid cholesterol and diminishes the emulsifying capacity. A monolayer of water prevents the denaturation of the protein [44]; when using S_A_, the result obtained was 72.33%. Both products of Na_G_ were proposed as additives with low cholesterol in products like muffins [4] and salad dressings [14]. The complex of egg yolk–mesquite gum with polyphenols can be used for the development of foods reduced in cholesterol, thereby helping avoid health problems like cardiovascular disease.

### 3.5. Microstructure Analysis

**Figure 3:**
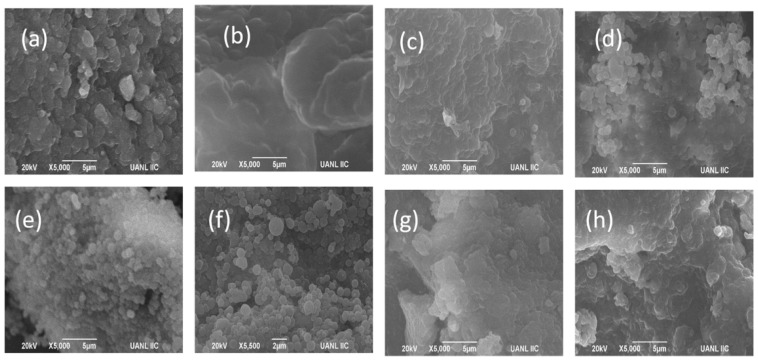
SEM photographs using different cholesterol extraction methods: (**a**) AG_1%_, (**b**) AG_3%_, (**c**) AG_10%_, (**d**) MG_1%_, (**e**) MG_3%_, (**f**) MG_10%_, (**g**) Na_G_, and (**h**) S_A_.

The microphotograph of Na_G_ (Figure 3g) shows irregular structures with small aggregates; it is probable these are HDL–phosvitin complexes linked by phosphocalcic bridges between the phosphate groups [45]. The microstructures of treatments with arabic gum (AG_1%_ AG_3%_, and AG_10%_; Figure 3a,b,c) show small aggregates, which are probably due to the interaction of the biopolymer carboxyl groups and the lipoproteins of egg yolk. In the microphotographs of different treatments with mesquite gum (MG_1%_, MG_3%_, and MG_10%_; Figure 3d,e,f), spherical structures within 2 to 5 μm of size were observed; it is probable that they correspond to the interaction between arabinogalactan proteins in mesquite gum and the lipoproteins of egg yolk [20].

The microphotograph of egg yolk shows an irregular structure because it is a complex system with several particles in suspension in a fluid that contains proteins. The microphotograph of egg yolk with acetone (S_A_) shows irregular structures with small aggregates.

### 3.6. Color Analysis

The values regarding the lightness (L*), redness (a*), and yellowness (b*) values are shown in Table 2. There are no significant differences for lightness L*, while a* and b* values show significant differences (*p* < 0.05). The Na_G_ shows low (Δ*E*) total color change, which we can state to be similar to egg yolk; this method removes less pigment than S_A_. The redness (a*) and yellowness (b*) values for Na_G_ were 1.7 and 22.73, respectively, greater than those reported for S_A_ (8.91 and 49.65, respectively). The color of egg yolk is attributed to carotenoids (xanthophylls, including lutein, zeaxanthin, β-cryptoxanthin, and β-carotene) [46]. We suggest that, if a large amount of cholesterol is removed, more carotenoids are also removed.

## 4. Conclusions

Separation efficiency of complex lipoproteins (HDLs)–mesquite gum shows a strong dependence on pH. The greatest cholesterol reduction was seen at pH 7.0. The amount of cholesterol removed was 97.24% using 1% mesquite gum (MG_1%_), and 93.26% using 1% arabic gum (AG_1%_). This is a consequence of the chemical composition in the chelate (mesquite gum or arabic gum) and yolk. The use of mesquite gum shows structural changes in the form of definite spheres with a low size in comparison with arabic gum, observed using SEM. The high removal of cholesterol contained in the egg yolk using mesquite gum or arabic gum reduced the primary emulsifying capacity of the egg yolk. The use of mesquite gum to remove cholesterol is an alternative method that does not require organic solvents. The use of 3% mesquite gum removed 12.83% more cholesterol than the same concentration of arabic gum. The complex obtained can be used in the development of functional foods reduced in cholesterol.

## Figures and Tables

**Table 1:** Cholesterol removal, efficiency, and emulsifying capacity of the different methods.

Method	% Cholesterol Reduction (pH 7)	% Cholesterol Reduction (pH 3)	% Efficiency (pH 7)	% Efficiency (pH 3)	Emulsifying Capacity (% *w*/*w*)
Na_G_	51.43 ± 1.86 ^e^	−	15.48 ± 1.31 ^d^	−	79.52 ± 2.01 ^b^
AG_1%_	93.26 ± 0.55 ^ab^	74.74 ± 1.10 ^ab^	29.72 ± 0.29 ^c^	3.72 ± 0.67 ^d^	63.57 ± 1.43 ^d^
AG_3%_	83.85 ± 3.80 ^c^	56.34 ± 0.57 ^b^	44.17 ± 3.35 ^ab^	4.38 ± 0.44 ^d^	−
AG_10%_	89.93±2.31 ^b^	86.33 ± 1.45 ^a^	37.37 ± 1.88 ^b^	16.42 ± 0.84 ^bc^	−
MG_1%_	97.24 ± 1.81 ^a^	87.75 ± 1.25 ^a^	13.50 ± 0.5 ^de^	12.14 ± 0.41 ^c^	62.95 ± 0.84 ^d^
MG_3%_	96.68±1.01 ^a^	92.55 ± 1.20 ^a^	14.85 ± 1.06 ^de^	19 ± 2.14 ^b^	−
MG_10%_	96.60 ± 2.04 ^a^	80.31 ± 2.30 ^a^	11.17 ± 0.72 ^e^	46.54 ± 3.38 ^a^	−
S_A_	64.15 ± 1.29 ^d^	−	44.51 ± 0.7 ^a^	−	72.33 ± 1.02 ^c^
Egg yolk	-	−	−	−	85.06 ± 0.051 ^a^

Note: Differing letters within a column are significantly different (*p* < 0.05) ± standard deviation (*n* = 3). Na_G_—physical separation of granules; AG—arabic gum; MG—mesquite gum; S_A_—solvent extraction.

**Table 2:** Color measurements of different methods for cholesterol reduction.

Method	L*	a*	b*	Δ*E*
Egg yolk	56.48 ± 0.01 ^a^	19.98 ± 0.01 ^e^	53.57 ± 0.05 ^d^	−
AG_1%_	79.76 ± 0.09 ^d^	4.7 ± 0.03 ^b^	35.29 ± 0.23 ^b^	33.32 ± 0.18 ^b^
AG_3%_	75.13 ± 3.94 ^bc^	6.73 ± 1.74 ^c^	41.29 ± 5.04 ^c^	26.03 ± 6.13 ^a^
AG_10%_	73.41 ± 0.01 ^b^	1.95 ± 0.00 ^a^	21.58 ± 0.05 ^a^	40.44 ± 0.08 ^c^
MG_1%_	75.12 ± 0.01 ^bc^	1.99 ± 0.01 ^a^	18.63 ± 0.01 ^a^	43.50 ± 0.05 ^c^
MG_3%_	81.92 ± 0.02 ^d^	1.43 ± 0.01 ^a^	19.28 ± 0.03 ^a^	46.56 ± 0.00 ^c^
MG_10%_	78.73 ± 0.04 ^cd^	4.64 ± 0.02 ^b^	33.21 ± 0.08 ^b^	33.84 ± 0.03 ^b^
S_A_	82.47 ± 0.01 ^d^	1.70 ± 0.01 ^a^	22.73 ± 0.02 ^a^	42.99 ± 2.20 ^c^
Na_G_	79.55 ± 0.01 ^d^	8.91 ± 0.01 ^d^	49.65 ± 0.01 ^d^	25.89 ± 0.01 ^a^

Note: Letters varying within a column are significantly different (*p* < 0.05) ± standard deviation (*n* = 3). L*—lightness; a*—redness; b*—yellowness; Δ*E*—total color change.

## References

[1] Sarika P., Pavithran A., James N.R. (2015). Cationized gelatin/gum arabic polyelectrolyte complex: Study of electrostatic interactions. Food Hydrocolloids.

[2] Islam A., Phillips G., Sljivo A., Snowden M., Williams P. (1997). A review of recent developments on the regulatory, structural and functional aspects of gum arabic. Food Hydrocolloids.

[3] Miranda J.M., Anton X., Valbuena C.R., Saavedra P.R., Rodríguez J.A., Lamas A., Franco C.M., Cepeda A. (2015). Egg and egg-derived foods: Effects on human health and use as functional foods. Nutrients.

[4] Marcet I., Collado S., Paredes B., Díaz M. (2015). Rheological and textural properties in a bakery product as a function of the proportions of the egg yolk fractions: Discussion and modelling. Food Hydrocolloids.

[5] Sun Y., Yang H., Zhong X., Zhang L., Wang W. (2011). Ultrasonic-assisted enzymatic degradation of cholesterol in egg yolk. Innovative Food Sci. Emerg. Technol..

[6] Lordan R., Tsoupras A., Mitra B., Zabetakis I. (2018). Dairy fats and cardiovascular disease: Do we really need to be concerned?. Foods.

[7] Scicchitano P., Cameli M., Maiello M., Modesti P.A., Muiesan M.L., Novo S., Palmiero P., Saba P.S., Pedrinelli R., Ciccone M.M. (2014). Nutraceuticals and dyslipidaemia: Beyond the common therapeutics. J. Funct. Foods.

[8] Quesada H., Díaz S., Pajuelo D., Fernández A., Garcia S., Pujadas G., Salvadó M.J., Arola L., Bladé C. (2012). The lipid-lowering effect of dietary proanthocyanidins in rats involves both chylomicron-rich and VLDL-rich fractions. Br. J. Nutr..

[9] Qin Y., Xia M., Ma J., Hao Y., Liu J., Mou H., Cao L., Ling W. (2009). Anthocyanin supplementation improves serum LDL- and HDL-cholesterol concentrations associated with the inhibition of cholesteryl ester transfer protein in dyslipidemic subjects. Am. J. Clin. Nutr..

[10] Trejo J.L. (2010). Establecimiento de un cultivo de células en suspensión de *Prosopis laevigata* (Humb. & Bonpl. ex Willd.) M. C. Johnst. para la producción de goma de mezquite. Ph.D. Thesis.

[11] Warren M., Brown H., Davis D. (1988). Solvent extraction of lipid components from egg yolk solids. J. Am. Oil Chem. Soc..

[12] Borges S., Martucci E., Müller C. (1996). Optimization of the extraction of cholesterol from dehydrated egg yolk using acetone. LWT-Food Sci. Technol..

[13] Paraskevopoulou A., Kiosseoglou V. (1997). Texture profile analysis of heat-formed gels and cakes prepared with low cholesterol egg yolk concentrates. J. Food Sci..

[14] Laca A., Sáenz M., Paredes B., Díaz M. (2010). Rheological properties, stability and sensory evaluation of low-cholesterol mayonnaises prepared using egg yolk granules as emulsifying agent. J. Food Eng..

[15] Alonso L., Fox P., Calvo M.V., Fontecha J. (2018). Effect of beta cyclodextrin on the reduction of cholesterol in ewe´s milk manchego cheese. Molecules.

[16] Jeong H., Sun H., Chogsom C., Kwak H. (2014). Cholesterol removal from whole egg by crosslinked β-cyclodextrin. Asian-Australasian J. Anim. Sci..

[17] Chiu S.H., Chung T.W., Giridhar R., Wu W.T. (2004). Immobilization of β-cyclodextrin in chitosan beads for separation of cholesterol from egg yolk. Food Res. Inter..

[18] García E.E., Reis J.S., Minim L.A., Freitas J. (2007). Cholesterol removal in liquid egg yolk using high methoxyl pectins. Carbohydr. Polym..

[19] Hsieh R.J., Snyder D.P., Ford E.W. (1994). Method for Removing Cholesterol and Fat from Egg Yolk by Chelation and Reduced-Cholesterol Egg Product. U.S. Patent.

[20] López Y.L., Goycoolea F.M., Valdez M.A., Calderón A.M. (2006). Goma de mezquite: Una alternativa de uso industrial. Interciencia.

[21] Pérez J., Barrios E., Róman A., Pedroza R. (2011). Interacción goma de mezquite-quitosano en la interfase y su influencia en la estabilidad de emulsiones múltiples W1/O/W2. Revista Mexicana de Ingeniería Química.

[22] Moreno M.B., Sánchez M. (2016). Mesquite gum as a novel reducing and stabilizing agent for modified tollens synthesis of highly concentrated Ag nanoparticles. Materials.

[23] Vernon E.J., Sherman P. (1980). Rheological properties and applications of mesquite tree (prosopis Juliflora) gum. 1. Rheologycal properties of aqueous mesquite gum solutions. J. Texture stud..

[24] Liu K.S. (1994). Preparation of fatty acid methyl esters for gas-chromatographic analysis of lipids in biological materials. J. Am. Oil Chem. Soc..

[25] García E., Higuera I., Espinosa H. (2017). Design of fish oil-in-water nanoemulsion by microfluidization. Innovative Food Sci. Emerg. Technol..

[26] Navidghasemizad S., Temelli F., Wu J. (2015). Phase separation behavior of egg yolk suspensions after anionic polysaccharides addition. Carbohydr. Polym..

[27] McClements D.J. (2016). Characterization of emulsion properties. Food Emulsions: Principles, Practice, and Technique.

[28] Valverde D., Laca A., Estrada L.N., Paredes B., Rendueles M., Díaz M. (2016). Egg yolk and egg yolk fractions as key ingredient for the development of a new type of gels. Int. J. Gastronomy Food Sci..

[29] Santipanichwong R., Suphantharika M. (2007). Carotenoids as colorants in reduced-fat mayonnaise containing spent brewer’s yeast β-glucan as a fat replacer. Food Hydrocolloids.

[30] Laca A., Paredes B., Rendueles M., Díaz M. (2014). Egg yolk granules: Separation, characteristics and applications in food industry. LWT-Food Sci. Technol..

[31] Strixner T., Kulozik U. (2013). Continuous centrifugal fractionation of egg yolk granules and plasma constituents influenced by process conditions and product characteristics. J. Food Eng..

[32] Martucci E., Borges S. (1997). Extraction of cholesterol from dehydrated egg yolk with acetone: Determination of the practical phase equilibrium and simulation of the extraction process. J. Food Eng..

[33] Puertas G., Vázquez M. (2018). Advances in techniques for reducing cholesterol in egg yolk: A review. Crit. Rev. Food Sci. Nutr..

[34] Gils P.S., Ray D., Sahoo P.K. (2010). Designing of silver nanoparticles in gum arabic based semi-IPN hydrogel. Int. J. Biol. Macromol..

[35] Navidghasemizad S., Temelli F., Wu J. (2014). Physicochemical properties of leftover egg yolk after livetins removal. LWT-Food Sci. Technol..

[36] Beristain C.I., Azuara E., Garcia H.S., Vernon E.J. (1996). Kinetic model for water/oil absorption of mesquite gum (*Prosopis juliflora*) and gum arabic (*Acacia senegal*). Int. J. Food Sci. Technol..

[37] Zuidam N.C., Nedovic V.A. (2010). Encapsulation Technologies for Active Food Ingredients and Food Processing.

[38] Strixner T., Sterr J., Kulozik U., Gebhardt R. (2014). Structural study on hen-egg yolk high density lipoprotein (HDL) granules. Food Biophys..

[39] Anton M. Recent Advances Concerning the Functional Properties of Egg Yolk Low-Density Lipoproteins. Proceedings of the EPC Proceedings of 12th European Poultry Conference.

[40] Turgeon S.L., Schmitt C., Sanchez C. (2007). Protein–polysaccharide complexes and coacervates. Curr. Opin. Colloid Interface Sci..

[41] De Kruif C., Tuinier R. (2001). Polysaccharide protein interactions. Food Hydrocolloids.

[42] Samant S., Singhal R., Kulkarni P., Rege D. (1993). Protein-polysaccharide interactions: A new approach in food formulations. Int. J. Food Sci. Technol..

[43] Orozco J., Cruz F., Ponce E., Vernon E. (2003). Mesquite gum: Fractionation and characterization of the gum exuded from Prosopis laevigata obtained from plant tissue culture and from wild trees. Carbohydr. Polym..

[44] Beyer J.D. (1991). The Development of a Cholesterol-Reduced Egg Yolk Using Solvent Extraction. Ph.D. Thesis.

[45] Huopalahti R., Anton M., López-Fandiño R., Schade R. (2007). Bioactive Egg Compounds.

[46] Li-Chan E.C., Kim H.O. (2007). Structure and Chemical Composition of Eggs. Egg Bioscience and Biotechnology.

